# Pain Assessment and Analgesic Requirements after Sleeve Gastrectomy: A Comparison Study of Robotic versus Laparoscopic Approaches

**DOI:** 10.3390/jcm13175168

**Published:** 2024-08-30

**Authors:** Juan S. Barajas-Gamboa, Mohammed Sakib Ihsan Khan, Valentin Mocanu, Jerry T. Dang, Gustavo Romero-Velez, Terrence Lee St-John, Gabriel Diaz Del Gobbo, A. Daniel Guerron, Juan Pablo Pantoja, Carlos Abril, Javed Raza, John Rodriguez, Matthew Kroh, Ricard Corcelles

**Affiliations:** 1 Digestive Disease Institute, Cleveland Clinic Abu Dhabi, Abu Dhabi 112412, United Arab Emirates; jbarajasgamboa@gmail.com (J.S.B.-G.); leestjt@clevelandclinicabudhabi.ae (T.L.S.-J.); gabodelgobbo@hotmail.com (G.D.D.G.); guerrod@clevelandclinicabudhabi.ae (A.D.G.); pantojj@clevelandclinicabudhabi.ae (J.P.P.); abrilc@clevelandclinicabudhabi.ae (C.A.); razaj@clevelandclinicabudhabi.ae (J.R.); rodrigj2@clevelandclinicabudhabi.ae (J.R.); 2 College of Medicine and Health Sciences, Khalifa University of Science and Technology, Abu Dhabi 127788, United Arab Emirates; mohammed.khan@ku.ac.ae; 3 Digestive Disease Institute, Cleveland Clinic, Cleveland, OH 44195, USA; vmocanu@ualberta.ca (V.M.); dangj3@ccf.org (J.T.D.); romerog4@ccf.org (G.R.-V.); krohm@ccf.org (M.K.); 4 Cleveland Clinic Lerner College of Medicine, Case Western Reserve University, Cleveland, OH 44106, USA

**Keywords:** sleeve gastrectomy, pain assessment, numerical rating scale, pain management, robotic surgery, laparoscopic surgery

## Abstract

**Background:** Sleeve gastrectomy is the most common bariatric procedure worldwide, yet postoperative pain management remains a concern. This study aimed to compare pain medication usage and pain scores between laparoscopic sleeve gastrectomy (LSG) and robotic sleeve gastrectomy (RSG) patients, addressing the potential benefits of RSG in postoperative pain control. **Methods:** A retrospective review of our institutional bariatric surgery registry included 484 patients (435 LSG, 49 RSG) who underwent surgery between September 2015 and November 2020. Pain management medications, including opioid use converted to morphine milligram equivalents (MMEs), and Numerical Rating Scale (NRS) scores were analyzed postoperatively using mixed-effects models. **Results:** RSG patients reported lower pain scores in the initial 24 h post-surgery and received significantly lower doses of morphine equivalents compared to LSG patients (median 7.5 mg vs. 12.5 mg, *p* < 0.001). RSG procedures had longer operative times (122.5 ± 27.1 vs. 89.9 ± 30.5 min, *p* < 0.001) but a shorter average length of stay (2.24 ± 0.60 vs. 2.65 ± 1.49 days) compared to LSG. **Conclusions:** These findings suggest that RSG may lead to reduced immediate postoperative pain and lower opioid requirements compared to the laparoscopic approach, despite longer operative times. Further randomized controlled trials are needed to confirm these observations and evaluate long-term outcomes.

## 1. Introduction

The evolution of sleeve gastrectomy (SG) over recent decades has been marked by the development of new surgical techniques, transitioning from open to laparoscopic, and more recently, to robotic approaches. This progression is a testament to both technological advancements and an ongoing effort to tackle emerging medical challenges [1]. Currently, laparoscopic sleeve gastrectomy (LSG) is predominant in surgical practice. Nevertheless, the advent of innovative surgical platforms and the introduction of novel robotic systems are quickly leading to increased use of robotic SG (RSG) [2].

RSG has emerged as an alternative to LSG, potentially offering several advantages. These may include enhanced three-dimensional visualization, improved ergonomics for the surgeon, and increased dexterity with wristed instruments. Theoretically, these features could lead to more precise dissection and suturing, potentially resulting in reduced tissue trauma and improved hemostasis. Some studies have suggested that RSG might be associated with shorter hospital stays, lower complication rates, and potentially better pain control in the immediate postoperative period. However, the true benefits of RSG over LSG are still a subject of ongoing research and debate in the bariatric surgery community [3].

An expected benefit of robotic techniques is the potential for reduced postoperative pain—a concern following LSG [4]. Concurrent with the advancement of surgical techniques, the opioid crisis has precipitated the development of multimodal postoperative pain management protocols [5]. These protocols target various pain pathways to minimize nociception, which aims to decrease analgesic requirements, reduce side effects, and minimize reliance on opioids [4,5]. An example of this approach might be the concurrent use of opioids and non-opioid analgesics [6]. Nonetheless, research studies specifically evaluating pain management in RSG are limited, and comparative studies between RSG and LSG are particularly lacking, which perhaps has slowed down the routine adoption of the robotic platform [6].

Data from a retrospective study including 45 LSG patients indicated a significant incidence (44.4%) of severe postoperative pain within the initial 24 h post-surgery [7]. Parallel findings were observed in RSG by Saleh et al. [8]. Contemporary recommendations for managing post-LSG pain suggest a regimen that includes acetaminophen, non-steroidal anti-inflammatory drugs (NSAIDs), and, if required, cautious supplementation with opioids. Gabapentinoids, while part of some regimens, are advised against due to their adverse effect profiles [9].

Considering the high prevalence of postoperative pain following SG and the critical role of effective pain management, it is essential to meticulously evaluate pain in the postoperative period [7,8]. Such an assessment is a cornerstone in developing protocols aimed at enhancing patient experience and reducing opioid prescriptions [9]. Effective pain control is instrumental in mitigating patient distress, facilitating better recovery, and expediting rehabilitation [3,9]. Thus, the objective of this study was to assess the usage of pain medications after elective bariatric surgery and compare pain scores between LSG and RSG.

## 2. Materials and Methods

Study design and ethical approvals: This retrospective review was conducted between September 2015 and November 2020, with approval from the Research Ethical Committee at our institution (Approval Code: A-2017-029, Approval Date: 26 September 2017). All patients undergoing SG were included. Data describing postoperative pain assessment and pain management were prospectively collected and analyzed. 

Data collection and research protocol: Data were collected prospectively in an institutional registry and retrospectively reviewed, including demographic characteristics, surgical approach, operative times, history of consumption of pain medications for chronic pain management, perioperative pain medications, transverse abdominis plane (TAP) block administration, and postoperative pain medication prescriptions. 

Primary and Secondary outcomes: The aim of this study was to assess the usage of pain medications after surgery and compare pain scores between primary elective LSG and RSG.

Inclusion and Exclusion criteria: Patients older than 16 years and younger than 70 years undergoing either primary LSG or RSG for the management of obesity and metabolic disease were included. Patients undergoing revisional surgery or had previous foregut surgery were excluded. Additionally, patients with diabetic neuropathy and a history of opioid abuse were excluded. 

### 2.1. Patient Qualification for LSG and RSG

All patients included in this study met the standard criteria for bariatric surgery as per the American Society for Metabolic and Bariatric Surgery (ASMBS) guidelines. These criteria include a body mass index (BMI) ≥40 kg/m^2^, or BMI ≥ 35 kg/m^2^, with at least one obesity-related comorbidity. The decision between LSG and RSG was made based on a combination of factors:

- Patient preference: After thorough explanation of both procedures, patients were given the option to choose between LSG and RSG, provided there were no contraindications.
- Surgeon assessment: The operating surgeon evaluated each patient’s specific anatomical and clinical factors. For instance, patients with extreme obesity (BMI > 60 kg/m^2^) or those with complex abdominal surgical history were more likely to be recommended for RSG due to the potential benefits of enhanced visualization and precision.
- Equipment availability: The allocation of patients to LSG or RSG was also influenced by the availability of the robotic system on the day of surgery.
- Contraindications: Patients with specific contraindications to pneumoperitoneum or Trendelenburg position were not considered for RSG.
- Learning curve considerations: To ensure patient safety during the initial phase of robotic surgery implementation, patients with lower BMI and fewer comorbidities were preferentially selected for RSG.

### 2.2. Surgical Approach and Perioperative Management

All sleeve gastrectomies in this study, both laparoscopic and robotic, were performed by a team of six experienced bariatric surgeons. Each of these surgeons had completed more than 100 LSGs and at least 30 RSGs prior to the study period, ensuring a high level of proficiency in both techniques. The surgical approach (LSG or RSG) was determined based on patient factors, surgeon preference, and operating room availability.

To maintain consistency in perioperative management, all surgeons followed a standardized Enhanced Recovery After Surgery (ERAS) protocol. This protocol included preoperative patient education, carbohydrate loading, multimodal pain management, early mobilization, and early oral intake. Specifically, patients were encouraged to ambulate within 6 h post-surgery, clear liquids were initiated on postoperative day 0, and advancement to a full liquid diet occurred on postoperative day 1, barring any complications.

The surgical technique for both LSG and RSG followed a standardized approach, including the use of a 36 Fr bougie for sleeve calibration, beginning the gastric transection 4–6 cm proximal to the pylorus, and reinforcing the staple line with absorbable sutures. This standardization helped to minimize technique-related variability between surgeons and procedures.

### 2.3. Pain Management and Assessment

The administration of pain medications followed a standardized protocol under the supervision of a dedicated pain management team. This team consisted of anesthesiologists and specially trained nurses. Preoperative and intraoperative medications were administered by the anesthesiology team. Postoperative pain management in the post-anesthesia care unit (PACU) was overseen by PACU nurses in consultation with the anesthesiology team. Once patients were transferred to the ward, pain medication administration was managed by floor nurses, following the pain management protocol and in consultation with the surgical team when necessary. This multidisciplinary approach ensured consistent and appropriate pain management throughout the patient’s hospital stay.

Type of medications, number of medications, and doses used preoperatively, intraoperatively, and postoperatively for management of pain were evaluated and analyzed. Morphine Equianalgesic Dose for Fentanyl was reported, as well as Morphine Milligram Equivalents for Tramadol [8,10].

Postoperative assessment of pain was performed using the Numerical Rating Scale (NRS) scoring system [11]. This scale spans from 0 to 10, where 0 is no pain and 10 is the worst pain ever experienced. Evaluations were completed by trained registered nurses at defined intervals, which included in the post-anesthesia care unit (PACU), after 2, 4, 8, 12, 24, 48, and 72 h, at discharge, and 1 week after surgery.

### 2.4. TAP Block Administration

In cases where a transversus abdominis plane (TAP) block was utilized, it was performed by the anesthesiology team immediately after the induction of general anesthesia and before the surgical incision. The TAP block was administered bilaterally using an ultrasound-guided technique. A total of 20 mL of 0.25% bupivacaine was injected on each side, ensuring proper spread of the local anesthetic in the transversus abdominis plane. This timing allowed for the optimal analgesic effect to be in place before the start of the surgical procedure, potentially contributing to reduced intraoperative and immediate postoperative pain.

### 2.5. Pain Management Protocol

Our institution follows a standardized multimodal pain management protocol for all sleeve gastrectomy patients, whether undergoing LSG or RSG. This protocol was designed to optimize pain control while minimizing opioid use. The protocol consists of the following components:

Preoperative:

1. Patient education on pain expectations and management strategies;
2. Administration of acetaminophen 1000 mg PO;
3. Administration of celecoxib 200 mg PO (if not contraindicated).

Intraoperative:

1. Transversus abdominis plane (TAP) block: 20 mL of 0.25% bupivacaine bilaterally, administered under ultrasound guidance after induction of anesthesia (when applicable);
2. Dexamethasone 8 mg IV at induction (for anti-emetic and analgesic properties);
3. Fentanyl IV as needed, titrated to effect.

Immediate postoperative (PACU):

1. Fentanyl IV for breakthrough pain, administered in 25–50 mcg increments as needed;
2. Ondansetron 4 mg IV every 6 h as needed for nausea/vomiting.

Postoperative Ward (initiated once oral intake is tolerated):

1. Acetaminophen 1000 mg PO every 6 h (not to exceed 4000 mg in 24 h);
2. Ketorolac 30 mg IV every 6 h for the first 24 h, then transition to ibuprofen 600 mg PO every 6 h if needed;
3. Tramadol 50–100 mg PO every 6 h as needed for moderate pain;
4. Oxycodone 5–10 mg PO every 4–6 h as needed for severe pain.

Discharge medications:

1. Acetaminophen 500 mg PO every 6 h as needed;
2. Ibuprofen 600 mg PO every 6 h as needed;
3. Oxycodone 5 mg PO every 6 h as needed for severe pain, limited to a 3-day supply.

Throughout the hospital stay, pain was regularly assessed using the NRS as previously described. The protocol allowed for adjustments based on individual patient needs and responses, always aiming to use the lowest effective dose of opioids. This standardized protocol was followed for all patients in the study, ensuring consistency in pain management approach between the LSG and RSG groups.

### 2.6. Statistical Analysis

1. Descriptive statistics: Continuous variables were summarized using mean (standard deviation) for normally distributed data and median (range) for non-normally distributed data. Categorical variables were presented as numbers and percentages.
2. Pain score analysis: Due to substantial positive skew in the pain score distribution, scores were log-transformed to reduce skew. Mixed-effects models were employed to account for the repeated-measures nature of the data (multiple pain assessments per patient).
3. Time series modeling: Time was divided into period groupings aligned with scheduled pain assessments (e.g., immediately post-procedure, 2 h post, 4 h post, etc.). A proxy series was specified at the repeated-measures level to account for a non-parametric time trend.
4. Medication analysis: For each time period T pain medications administered after the prior pain assessment (T minus 1) but before the current assessment T were captured at the repeated-measures level with indicator variables. Pre-procedure and intraoperative pain medications were captured with surrogate series and were allowed to moderate the repeated-measures level intercept.
5. Comparative analysis: “Approach by time-period” interaction variables were specified to examine differences between LSG and RSG at each time period. The approach difference immediately post-procedure served as the reference when testing these interaction effects.
6. Interactions with associated *p*-values greater than 0.30 were removed from the model, producing the reduced model presented in this manuscript.
7. Additional calculations: The number of medications administered, mean dosages, and number of administrations of a given medication were calculated. Specific focus was given to intraoperative fentanyl and postoperative opioids, ketorolac, acetaminophen, and tramadol.
8. Significance level: A *p*-value <0.05 was considered statistically significant for all analyses.
9. Software: All analyses were performed using R (version 2.13 or higher, The R Foundation for Statistical Computing, Vienna, Austria).

## 3. Results

### 3.1. Patients’ Characteristics and Demographics

A total of 484 patients were included in this study, with 435 (89.8%) patients undergoing LSG and 49 (10.2%) receiving RSG. Of these, 267 (54.9) patients were female with a median age of 33.0 (16–70) years. The average initial body mass index (BMI) for all patients was 44.9 ± 7.3 kg/m^2^, with no significant difference between both groups. The most common comorbidities in this cohort are described in (Table 1).

### 3.2. Pain Management

**Preoperative:** As a part of standard institutional protocols, patients are subjected to receive immediate preoperative medications for pain management. There was a no significant difference (*p* = 0.23) between the administration of pain medications which occurred in 400 (92.2%) of the LSG and 47 (98%) of the RSG patients. Furthermore, celecoxib was administrated to 215 (49.5%) of LSG and 28 (58.3%) of RSG patients (*p* = 0.28). 

**Intraoperative findings:** Mean operative time was significantly higher for RSG with 122.5 ± 27.1 min and 89.9 ± 30.5 min for LSG (*p* < 0.001). There was a significant difference (*p* = 0.005) in the performance of TAP block, being conducted in 12.2% of LSG and 27% of RSG procedures. Anesthesia protocols included pain medications as well. The most frequent was fentanyl, which was used in 85% of the LSG patients and 78% of the RSG patients (*p* = 0.40). The average dose was 50 mcg in both groups. Acetaminophen was also a common pain medication used intraoperatively and was used in 58.5% of LSG patients and in 75% of RSG patients (*p* = 0.18) (Table 2).

Postoperative and post discharge: The average length of stay (LOS) after LSG was 2.65 ± 1.49 days, while the average LOS after RSG was 2.24 ± 0.60 days, (*p* = 0.064). In the RSG group, 91.8% received opioids, while 89.2% of the LSG group received them. Opioid medications that were administered were analyzed during the recovery period and discharge. There was no significant difference in the doses of fentanyl (*p* = 0.20), but there was a significant difference in morphine use (*p* < 0.001) between both cohorts after the procedures (Table 3). There was no significant difference in the doses of ketorolac (*p* = 0.078) and acetaminophen (*p* = 0.669) between both cohorts after discharge. Tramadol was administrated as needed in 55.3% after LSG and 50% after RSG (Table 4 and Table 5). Moreover, the number of pain medications administered was also assessed. In LSG, 8% of patients received two medications, 28% received three, and 64% received more than three medications, whereas in RSG, 10% received two medications, 37% received three, and 53% received more than three medications.

### 3.3. Pain Assessment

Postoperative and follow-up pain assessment: A larger portion of RSG patients reported ‘no pain’ (or a score of 0 on the NRS) in comparison to LSG patients during the study period. Comparing both groups, the percentage of patients who were pain free at discharge represented 67.3% in the RSG group and 64.1% in the LSG group (*p* = 0.561). At 1-week follow-up, the percentage of patients who were pain free was 18.4% in the RSG group and 21.1% in the LSG group (*p* = 0.652). The pain assessments during the different time points in both groups are described in (Table 6).

## 4. Discussion

In this study comparing RSG to LSG, we found three key findings: (1) RSG was associated with lower pain scores and reduced morphine equivalent requirements in the initial 24 h post-surgery, (2) no significant differences existed between procedures with respect to medication use or overall pain scores at discharge, and (3) RSG procedures had longer operative times but shorter average length of stay.

Our results show that RSG is associated with favorable early pain scores (<24 h) compared to LSG. In the initial 24 h following the procedure, a higher percentage of RSG patients consistently reported no pain (NRS score of 0) compared to LSG patients. This early advantage in pain management for RSG patients is likely attributable to the increased use of transversus abdominis plane (TAP) blocks in the RSG group (27% vs. 12.2%, *p* = 0.005). TAP blocks are known to provide effective analgesia in the immediate postoperative period, which aligns with our observations [7,8].

After the 24 h mark, the pain score trends between RSG and LSG patients became less distinct, with no statistically significant differences observed. By the time of discharge, RSG patients again reported a higher percentage with an NRS score of 0, but overall, no significant differences existed between procedures with respect to medication use or pain scores at discharge.

The medications administered for pain management intraoperatively and postoperatively showed similar patterns between groups, with a few exceptions. More patients in the LSG group used fentanyl and morphine during the recovery phase after surgery. As shown in Table 3 and Table 4, while the mean dose of opioids as part of intraoperative and postoperative medication was similar for both groups, RSG patients required lower doses of morphine equivalents in the early postoperative period.

Regarding operative characteristics, the mean operative time for RSG was significantly longer than LSG, but interestingly, the average length of stay (LOS) for RSG was shorter. This observation of longer operative times for RSG is consistent with findings from other studies [7,8]. The longer duration is likely due to the additional time required for docking and undocking the robot, as well as the current learning curve associated with robotic techniques.

The observation of less immediate postoperative pain in RSG patients despite longer operative times may seem counterintuitive at first glance. However, several factors could contribute to this finding. Firstly, the robotic system’s enhanced precision and control may result in less tissue manipulation and trauma during the procedure, potentially leading to reduced postoperative pain regardless of the longer operative time. Secondly, the improved ergonomics of the robotic system may allow for more meticulous hemostasis and closure, which could contribute to decreased postoperative pain. Additionally, the longer operative time in RSG is primarily due to the docking and undocking of the robot, rather than increased surgical manipulation time. Finally, it is worth noting that a higher proportion of RSG patients received transversus abdominis plane (TAP) blocks (27% vs. 12.2%, *p* = 0.005), which likely contributed to improved immediate postoperative pain control in this group. These factors, combined with the potential benefits of the robotic approach, may explain the reduced immediate postoperative pain despite longer operative times.

Previous studies have reported that in bariatric surgery, postoperative complications such as atelectasis, pneumonia due to respiratory splinting, and other lung complications are more frequent, and these complications might worsen patients’ sleep apnea/hypopnea syndrome [12,13]. Therefore, it is important to optimize preventive measures such as pain control and early mobilization [12]. In addition, higher rates of thromboembolic events have been reported in this population due to poor ambulation from pain [14]. Other studies have shown that inappropriate pain management increases the chances of postoperative readmission as well as longer hospital stays [15].

Other aspects associated with inappropriate pain management protocols include pain medication addiction. A study conducted by Hoehn et al. in 2019 concluded that bariatric surgery patients are at risk of chronic addiction to pain medications due to baseline conditions such as arthritis represented in knee and back pain [16]. As such, it is important to evaluate the use of pain medications (especially opioids) preoperatively, as that could have an impact on nociception, especially during times of an opioid epidemic. Chronic use of opioids may also influence the risk of postoperative respiratory depression [17]. Bariatric surgeons are attempting to decrease perioperative morbidity and streamline care. One way of accomplishing this is by decreasing opioid use and hospital stay [18].

In a retrospective analysis of same-day surgeries, Coley et al. observed that pain was the predominant reason for unexpected hospital admission or readmission post-procedure, reported in 120 patients, which accounts for 38% of patients [19]. Furthermore, several studies of patients undergoing a broad variety of surgery types have demonstrated that the presence and intensity of acute postoperative pain were significant predictive risk factors for the development of chronic pain [20,21].

In a study on postoperative pain in RSG conducted by Alper et al., there was no difference in immediate postoperative narcotic use for pain when compared to LSG [22], which is consistent with our study. A retrospective cohort study by Saleh et al. on 286 LSG and 82 RSG patients revealed no significant differences in opioid use intraoperatively, but opioids were prescribed in a significantly smaller proportion of RSG patients compared to LSG [8]. Furthermore, Saleh et al. and Pepper et al. in their studies noted a significantly longer operative time for RSG compared to LSG, which is consistent with the findings of this study [8,23].

Our study’s findings on length of stay (LOS) contribute to the ongoing debate in the literature regarding robotic versus laparoscopic sleeve gastrectomy. 

While a National Inpatient Study reported significantly higher LOS in the RSG group, our results align with a systematic review and meta-analysis that found shorter LOS after RSG [24]. This discrepancy underscores the complexity of evaluating surgical outcomes. Our research adds to the existing knowledge by demonstrating shorter LOS for RSG despite longer operative times, suggesting that the initial time investment in robotic procedures may be offset by faster postoperative recovery [25,26]. Furthermore, our detailed analysis of pain scores at various time points offers a more nuanced understanding of the postoperative pain experience in both procedures, an aspect not extensively explored in previous studies.

The limitations of this study include variable dosing regimens, variable methods of administration, and time points of pain assessments. Another important limitation is the use of TAP block in some patients, which could not be adjusted for but should not be responsible for differences in pain scores past 24 h. Furthermore, the small sample size has the potential for over- or under-estimation of effect. In addition, it is important to note that pain is a subjective sensation. What might feel like a 10 to an individual on the NRS could also feel like a 5 to another. Additionally, selection bias should be considered as different patients may undergo RSG versus LSG depending on their specific cases, further complicating direct comparisons between the two procedures. Consequently, it appears to be difficult to standardize these results.

An additional limitation of our study is the significant difference in the proportion of diabetic patients between the LSG and RSG groups (24.5% vs. 10.2%, *p* = 0.02). This disparity could potentially impact our pain assessment results due to the presence of diabetic neuropathy. Neuropathy, a common complication of diabetes, can alter pain perception, potentially leading to underreporting of pain in affected individuals [20]. Conversely, some studies suggest that diabetic patients may experience heightened pain sensitivity in certain contexts. The complex relationship between diabetes, neuropathy, and pain perception could introduce bias in our pain score comparisons [21]. While our study did not specifically assess for neuropathy, future research should consider screening for and stratifying results based on the presence of diabetic neuropathy. This approach would allow for a more nuanced understanding of how diabetes and its complications might influence postoperative pain experiences in bariatric surgery patients.

There is limited literature in this study field, particularly in SG patients. This is one of the few studies comparing postoperative opioid usage. It is one of the first studies to report pain scores between laparoscopic and robotic SG at different time points of 2, 4, 8, 12, 24, 48, and 72 h after surgery, at discharge, and 1 week after surgery. RSG is still gaining popularity; as such, more studies should be available to assess its benefits and disadvantages. Additionally, new data would also help surgeons to compare this approach to previous techniques. Being a newer technique, RSG reduces movement and torque at the abdominal wall, likely reducing postoperative pain compared to other surgical techniques. This study allowed us to assess the extent of postoperative pain and how it is managed by different patients. Given the subjectivity of the sensation of pain, it is difficult to standardize the prescription of pain medications. As such, patient education on the latter is important.

## 5. Conclusions

This study found that robotic sleeve gastrectomy (RSG) patients reported less immediate postoperative pain and required significantly lower doses of morphine equivalents compared to laparoscopic sleeve gastrectomy (LSG) patients in the early postoperative period (median 7.5 mg vs. 12.5 mg, *p* < 0.001). However, it is important to note that no significant differences existed between procedures with respect to medication use or overall pain scores at discharge. While RSG had longer operative times, it was associated with shorter hospital stays. These findings contribute to the ongoing discussion about the potential benefits and limitations of robotic approaches in bariatric surgery, particularly in terms of early postoperative pain management and recovery.

## Figures and Tables

**Table 1:** Patient’s characteristics and demographics.

	LSG (*n* = 435)	RSG (*n* = 49)	*p*-Value
Patient Factors			
Age (median, range)	29 (18–63)	33 (18–70)	0.06
Female (N, %)	239 (54.9)	28 (57.1)	0.77
Initial BMI (mean, SD)	45.0 (±7.4)	43.3 (±5.3)	0.11
Functional status			
Independent	435 (100)	49 (100)	-
Smoker (N, %)	92 (21.1)	17 (34.6)	0.03
ASA category			0.45
ASA 1–2	128 (29.4)	18 (36.7)	
ASA 3	305 (70.1)	31 (63.3)	
ASA 4–5	2 (0.5)	0 (0.0)	
Comorbidities (N, %)			
Diabetes	107 (24.5)	5 (10.2)	0.02
OSA	135 (31.0)	15 (30.6)	0.95
HTN	134 (30.8)	14 (28.5)	0.87
GERD	132 (30.3)	7 (14.2)	0.01
COPD	3 (0.6)	0 (0.0)	0.56
DLD	183 (42.0)	22 (44.8)	0.75
Chronic steroids	23 (5.2)	0 (0.0)	0.14
Renal insufficiency	19 (4.3)	2 (4.0)	0.92
Dialysis	4 (0.9)	0 (0.0)	0.50
Prior VTE	6 (1.3)	1 (2.0)	0.71
Therapeutic anticoagulation	15 (3.4)	0 (0.0)	0.37
Prior MI	15 (3.4)	1 (2.0)	0.60

Abbreviations: BMI—body mass index; ASA—American Society of Anesthesiologists; OSA—obstructive sleep apnea; HTN—hypertension; GERD—gastroesophageal reflux disease; COPD—chronic obstructive pulmonary disease; DLD—dyslipidemia; LSG—laparoscopic sleeve gastrectomy; RSG—robotic sleeve gastrectomy; VTE—venous thromboembolism; MI—myocardial infarction.

**Table 2:** Pain medications administrated as part of the anesthesia protocols.

	LSG	RSG	*p*-Value
Mean operative time (minutes)	89.9 ± 30.5	122.5 ± 27.1	<0.001
Transverse abdominis plane (TAP) block performed (%)	53 (12.2%)	13 (27%)	0.005
Use of fentanyl IV (%)	219 (50.5%)	29 (60.4%)	0.404
Mean fentanyl dose (mcg)	50	50	-
Morphine Equivalent Daily Dose (mg/day)	2880	2880	-
Use of acetaminophen 1000 mg (%)	254 (58.5%)	36 (75%)	0.188
Use of other analgesic medications (%)	199 (45.7%)	6 (12.7%)	<0.001

**Table 3:** Use of opioids during recovery.

Medication	Mean Dose LSG Cohort	# of Pts LSG	Mean Dose RSG Cohort	# of Pts RSG	Time Period
Fentanyl IV	50 mcg	305 (70.3%)	50 mcg	33 (68.8%)	48 h
MEDD using fentanyl IV	2880 mg/day	305 (70.3%)	2880 mg/day	33 (68.8%)	24 h
Morphine IV	9.45 mg	226 (52.1%)	10 mg	21 (43.8%)	48 h
MEDD using fentanyl IV combined with Morphine IV	2889.45 mg/day	-	2890 mg/day	-	24 h

Abbreviations: MEDD: Morphine Equivalent Daily Dose.

**Table 4:** Postoperative pain medications upon admission.

Medication	Mean Dose in LSG Cohort	Mean Dose in RSG Cohort	Mean # of Doses LSG	Mean # of Doses RSG
Ketorolac IV	30 mg	30 mg	6.74 ± 2.20	7.55 ± 1.01
Acetaminophen IV	994.1 mg	994.7 mg	5.97 ± 1.57	6.04 ± 0.99

**Table 5:** Postoperative pain medications in admission.

Medication	Mean # of Doses LSG	# of Patients in LSG (%)	Mean # of Doses RSG	# of Patients in RSG (%)
Tramadol 100 mg = 10 MEDD	2.64 ± 1.78	235 (54.1%)	1.82 ± 1.03	24 (50%)
Tramadol 50 mg = 5 MEDD	2.40 ± 1.52	5 (1.2%)	0	0 (0%)

Abbreviations: MEDD: Morphine Equivalent Daily Dose.

**Table 6:** Numerical Rating Scale scores after sleeve gastrectomy at multiple time points, shaded columns—Post-operative pain medications in admission, N/A—not available.

	Score											
	0	1	2	3	4	5	6	7	8	9	10	N/A
	%	%	%	%	%	%	%	%	%	%	%	%
Immediately after surgery												
LSG	41.8	3.9	6.2	7.1	5.7	6.7	6.7	7.4	4.6	1.6	1.6	6.7
RSG	59.2	8.2	2.0	2.0	6.1	6.1	8.2	2.0	4.1	0	0	2.0
2 h after surgery												
LSG	27.1	6.9	21.4	13.6	8.3	6.2	3.4	2.3	3.2	0.5	0.7	6.4
RSG	32.7	4.1	16.3	12.2	6.1	4.1	4.1	10.2	4.1	0	0	6.1
4 h after surgery												
LSG	31.3	2.3	11.7	7.6	6.7	9.0	6.0	4.4	4.4	0.5	0.7	15.6
RSG	32.7	2.0	14.3	6.1	14.3	4.1	4.1	4.1	2.0	0	0	16.3
8 h after surgery												
LSG	45.5	2.5	6.7	7.6	6.2	6.7	4.1	3.0	1.1	0.9	0	15.6
RSG	51.0	2.0	10.2	2.0	4.1	2.0	2.0	4.1	2.0	0	0	20.4
12 h after surgery												
LSG	52.9	1.6	6.7	7.1	5.5	3.9	3.9	2.5	0.9	0.2	0.2	14.5
RSG	63.3	2.0	2.0	6.1	6.1	2.0	2.0	2.0	0	0	0	14.3
24 h after surgery												
LSG	51.7	2.3	8.7	5.3	6.4	5.1	3.7	1.6	0.7	0.5	0.7	13.3
RSG	67.3	2.0	6.1	2.0	4.1	4.1	0	0	0	0	0	14.3
48 h after surgery												
LSG	24.8	0.7	3.9	3.9	4.4	2.3	1.4	0.9	0	0.2	0.2	57.2
RSG	16.3	0	2.0	0	0	4.1	0	0	0	0	0	77.6
72 h after surgery												
LSG	8.0	0.2	1.1	0.2	0.2	0.7	0.2	0.9	0	0	0	88.3
RSG	2.0	0	0	0	0	0	0	0	0	0	2.0	95.9
At discharge												
LSG	64.4	1.8	8.5	6.2	8.3	3.2	2.8	3.0	0.5	0	0.2	1.1
RSG	67.3	4.1	10.2	0.0	2.0	10.2	2.0	2.0	0	2.0	0	0

## Data Availability

The data that support the findings of this study are available from the corresponding author upon reasonable request.
